# Aging and Comorbidities in Acute Pancreatitis II.: A Cohort-Analysis of 1203 Prospectively Collected Cases

**DOI:** 10.3389/fphys.2018.01776

**Published:** 2019-04-02

**Authors:** Zsolt Szakács, Noémi Gede, Dániel Pécsi, Ferenc Izbéki, Mária Papp, György Kovács, Eszter Fehér, Dalma Dobszai, Balázs Kui, Katalin Márta, Klára Kónya, Imre Szabó, Imola Török, László Gajdán, Tamás Takács, Patrícia Sarlós, Szilárd Gódi, Márta Varga, József Hamvas, Áron Vincze, Andrea Szentesi, Andrea Párniczky, Péter Hegyi

**Affiliations:** ^1^Institute for Translational Medicine, University of Pécs, Medical School, Pécs, Hungary; ^2^János Szentágothai Research Centre, University of Pécs, Pécs, Hungary; ^3^First Department of Internal Medicine, Szent György University Hospital in Fejér County, Székesfehérvár, Hungary; ^4^Department of Internal Medicine, Division of Gastroenterology, Faculty of Medicine, University of Debrecen, Debrecen, Hungary; ^5^First Department of Medicine, University of Szeged, Szeged, Hungary; ^6^University of Medicine and Pharmacy of Târgu Mures, Târgu Mures, Romania; ^7^Division of Gastroenterology, First Department of Medicine, University of Pécs, Medical School, Pécs, Hungary; ^8^Emergency County Hospital Targu Mures, Târgu Mureş, Romania; ^9^Békés Megyei Központi Kórház Dr. Réthy Pál Tagkórház Hospital, Gastroenterology, Békéscsaba, Hungary; ^10^Bajcsy-Zsilinszky Hospital, Budapest, Hungary; ^11^Heim Pál National Institute of Pediatrics, Budapest, Hungary; ^12^Division of Translational Medicine, First Department of Medicine, University of Pécs, Medical School, Pécs, Hungary; ^13^Hungarian Academy of Sciences, Momentum Gastroenterology Multidisciplinary Research Group, University of Szeged, Szeged, Hungary

**Keywords:** acute pancreatitis, comorbidities, mortality, severity, length of hospitalization, complications, prediction, Charlson Comorbidity Index

## Abstract

**Introduction:** Our meta-analysis indicated that aging influences the outcomes of acute pancreatitis (AP), however, a potential role for comorbidities was implicated, as well. Here, we aimed to determine how age and comorbidities modify the outcomes in AP in a cohort-analysis of Hungarian AP cases.

**Materials and Methods:** Data of patients diagnosed with AP by the revised Atlanta criteria were extracted from the Hungarian Registry for Pancreatic Patients. Outcomes of interest were mortality, severity, length of hospitalization, local, and systemic complications of AP. Comorbidities were measured by means of Charlson Comorbidity Index (CCI) covering pre-existing chronic conditions. Non-parametric univariate and multivariate statistics were used in statistical analysis. Odds ratios (ORs) with 95% confidence intervals (CIs) were calculated.

**Results:** A total of 1203 patients from 18 centers were included. Median age at admission was 58 years (range: 18–95 years), median CCI was 2 (range: 0–10). Only severe comorbidities (CCI ≥ 3) predicted mortality (OR = 4.48; CI: 1.57–12.80). Although severe comorbidities predicted AP severity (OR = 2.10, CI: 1.08–4.09), middle (35–64 years) and old age (≥65 years) were strong predictors with borderline significance, as well (OR = 7.40, CI: 0.99–55.31 and OR = 6.92, CI: 0.91–52.70, respectively). Similarly, middle and old age predicted a length of hospitalization ≥9 days. Interestingly, the middle-aged patients (35–64 years) were three times more likely to develop pancreatic necrosis than young adults (OR = 3.21, CI: 1.26–8.19), whereas the old-aged (≥65 years) were almost nine times more likely to develop systemic complications than young adults (OR = 8.93, CI: 1.20–66.80), though having severe comorbidities (CCI ≥ 3) was a predisposing factor, as well.

**Conclusion:** Our results proved that both aging and comorbidities modify the outcomes of AP. Comorbidities determine mortality whereas both comorbidities and aging predict severity of AP. Regarding complications, middle-aged patients are the most likely to develop local complications; in contrast, those having severe comorbidities are prone to develop systemic complications. Studies validating the implementation of CCI-based predictive scores are awaited.

## Introduction

The annual incidence of AP ranges from 10 to 100 cases per 100,000 persons (Roberts et al., 2013), showing an increasing tendency throughout the past decades (Yadav and Lowenfels, 2013). Multiple theories have been proposed to explain the increment: better diagnostics (e.g., general access to the measurement of pancreatic enzymes) (Yadav et al., 2011), lifestyle factors (e.g., obesity, alcohol consumption, and tobacco use) (Alsamarrai et al., 2014; Samokhvalov et al., 2015) as well as aging of the population (Spanier et al., 2013) have been implicated.

Aging not only increases the risk of AP (Yadav and Lowenfels, 2013) but also may change the clinical course of it, resulting in higher mortality (Fan et al., 1988; Spanier et al., 2013) and longer hospitalization (Murata et al., 2011; McNabb-Baltar et al., 2014), thereby increases the cost for care in the elderly (Fagenholz et al., 2007; Murata et al., 2012). Accordingly, widely accepted predictive scores and severity indices, such as Ranson criteria (age > 55 years) (Ranson et al., 1974), APACHE II (age > 44 years) (Larvin and McMahon, 1989), and BISAP (age > 60 years) (Wu et al., 2008) consider age as a risk factor of worse clinical outcomes, where the potential impact of comorbidities is omitted from these.

Risk of morbidities increases with age (Vasilopoulos et al., 2014). Since the average age of AP onset is around 55–70 years (Yadav and Lowenfels, 2013; Hamada et al., 2014), most AP patients are exposed to the burden of comorbidities (Murata et al., 2015). Sporadic studies reported on how comorbidities affect the outcomes of AP: they increases mortality (Singla et al., 2009; Murata et al., 2011, 2015; Akshintala et al., 2013; McNabb-Baltar et al., 2014) and the length of hospital stay, as well (Murata et al., 2011, 2015; Francisco et al., 2013). However, the predictive role of comorbidities is underutilized regarding AP severity and the development of complications.

Results of the meta-analysis by Marta et al. (under revision) published in the previous issue of Frontiers Science suggested that both mortality and severity of AP are age-dependent, but age alone does not explain the increment of mortality in the elderly. This increment might be attributed to comorbidities, as shown in Figure 11 by Marta et al. (under revision). These findings inspired us to conduct a cohort-analysis of AP cases to provide a comprehensive assessment on how aging and comorbidities alter outcomes of AP including mortality, severity, LOH, and complications; and to decide whether the burden of aging or comorbidities is decisive for determining hard outcomes.

## Materials and Methods

### Population

We extracted data from the Hungarian Registry for Pancreatic Patients (AP Registry) established in 2011 by the Hungarian Pancreatic Study Group in order to advance clinical care and research in Pancreatology (Parniczky et al., 2016). AP Registry contains data on consecutive cases of AP attending several Hungarian centers between 2011 and 2017. Accuracy of data recorded is secured by a four-level quality check system involving both medical administrative personnel and gastroenterologist specialists.

### Comorbidities

Registry forms of AP cases involve an admission form (A form) and follow-up forms (B-forms) covering the entire hospital stay, as well as the de-identified electronic discharge files. All files were carefully reviewed by an author with a medical degree to aggregate CCI (Charlson et al., 1987) with the International Classification of Diseases 9/10 coding algorithm (Quan et al., 2005). No search engines were used when reviewing charts. CCI items were dedicated to rating common chronic pre-existing diseases along 19 health-related (groups of) conditions. Every CCI item has a weight according to the severity of comorbidities covered (Charlson et al., 1987). CCI of each case was calculated by compiling the weighted items. Earlier studies proved that CCI is an effective predictor of hard outcomes in several acute and chronic conditions (Ng et al., 2013; Frenkel et al., 2014; Marventano et al., 2014).

### Eligibility Criteria

To be included in analysis, the following criteria should be met:

(1) Diagnosis of AP (“Two out of three”) (Working Group Iap/Apa Acute Pancreatitis Guidelines, 2013):
  (i) Abdominal pain
  (ii) Serum amylase and/or lipase greater than three times the upper normal limit
  (iii) Characteristic findings on abdominal cross-sectional imaging
(2) Age ≥ 18 years
(3) Available history for CCI (Charlson et al., 1987)

### Outcomes

Our AP-related outcomes included in-hospital mortality, severity, LOH, local complications (including peripancreatic fluid collections, pseudocysts, and pancreatic necrosis), and organ failure (including respiratory, renal, and cardiac failure).

### Ethical Approval

AP Registry has been approved by Scientific and Research Ethics Committee of the Medical Research Council, Hungary (22254-1/2012/EKU). All subjects gave written informed consent in accordance with the Declaration of Helsinki.

### Statistical Analysis

An expert biostatistician carried out the analysis with SPSS 19.0.0 (IBM Analytics, United States). Case numbers and percentages were calculated for categorical variables, medians with 25% and 75% quartiles (Q_1_ and Q_3_, respectively) and ranges were computed for numerical variables in descriptive analysis (due to non-normal distribution of data indicated by the Kolmogorov–Smirnov test). In all analysis, a probability (*p*) < 0.05 indicated a significant difference, whereas a *p*-value between 0.05 and 0.10 indicated borderline significance.

Representativeness of study population was tested by binomial, one sample median, and Goodness-of-fit χ2 tests.

In univariate analysis, Spearmann’s rho was calculated to explore correlations between age, CCI, and LOH. ORs with 95% CIs were calculated from 2 × 2 tables. If OR was not calculable, association were investigated with χ^2^ - or Fisher’s tests.

In multivariate analysis, binary logistic and multinominal regressions were used to investigate the joint effect of age categories and CCI categories or that of age categories and individual comorbidities. We used a three-level age-stratification (young-aged between 18 and 34 years of age, middle-aged between 35 and 64 years of age, and old-aged ≥ 65 years of age) and a four-level comorbidity stratification (none if CCI = 0, mild if CCI = 1, moderate if CCI = 2, and severe if CCI ≥ 3).

## Results

### Demography

AP Registry contained 1241 cases, of them 1203 (96.9%) from 18 centers were eligible for inclusion. Demography of study population and that of AP Registry are presented in Table 1 and Supplementary Appendix 1, respectively. Distribution of sites of recruitment is presented in Supplementary Appendix 1. Study population proved to be representative to that of AP Registry regarding demography and disease outcomes (*p* > 0.05 for all variables analyzed) (Supplementary Appendices 2, 9). Data quality for all variables was >99% in study population (Supplementary Appendix 3).

**Table 1:** Demography of study population including a total of 1203 cases of acute pancreatitis (AP).

Age, median (Q_1_–Q_3_)	58 (44–70)
Sex, n_male_ (%_male_)	670 (55.7)
Etiology (pure)	
Biliary, n (%)	528 (43.9)
Alcoholic, n (%)	269 (22.4)
Hypertriglyceridemic, n (%)	69 (5.7)
Mortality, n (%)	28 (2.3)
Severity of pancreatitis	
Mild, n (%)	825 (68.6)
Moderate, n (%)	313 (26.0)
Severe, n (%)	65 (5.4)
Length of hospitalization, median (Q_1_–Q_3_)	9 (7–14)
Local complications, n (%)	358 (29.8)
Fluid collection, n (%)	303 (25.2)
Pseudocyst, n (%)	120 (10.0)
Necrosis, n (%)	111 (9.2)
Systemic complications, n (%)	92 (7.7)
Respiratory failure, n (%)	55 (4.6)
Heart failure, n (%)	19 (1.6)
Renal failure, n (%)	33 (2.7)
Charlson Comorbidity Index, median (Q_1_–Q_3_)	2 (0–2)
Severity of comorbidities	
No comorbidities, n (%)	444 (36.9)
Mild comorbidities, n (%)	345 (28.7)
Moderate comorbidities, n (%)	190 (15.8)
Severe comorbidities, n (%)	224 (18.6)

*Continuous variables are presented in median with quartiles (Q_1_–Q_3_), categorical variables are presented in frequencies (n) with percentages of total (%).*

### Association Between Aging and Comorbidities in AP

#### Aging Strongly Influences the Outcomes of AP in Univariate Models

**FIGURE 1:**
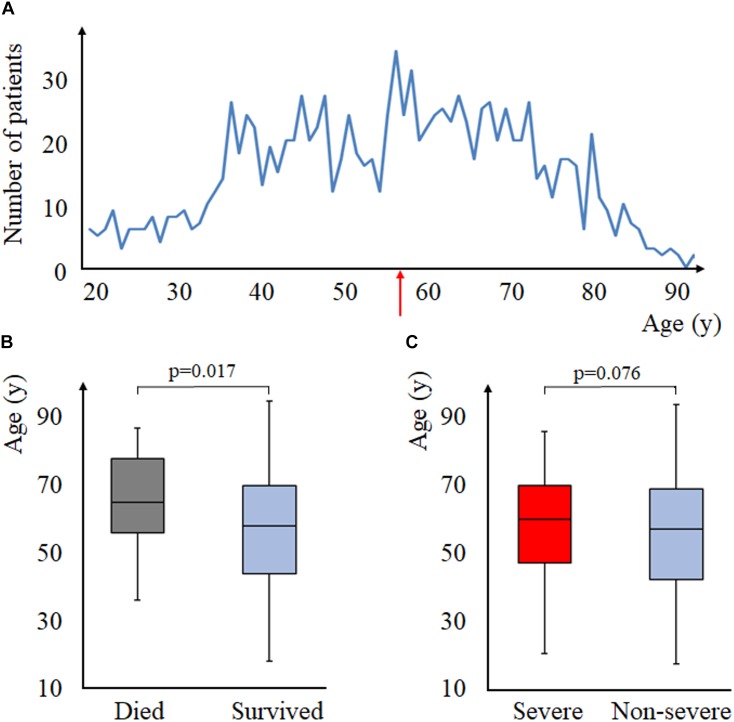
Aging and acute pancreatitis (AP). **(A)** age-distribution of the study population, the red arrow indicates the median age of the population (that is, 58 years of age). **(B)** mortality and age (Mann–Whitney test). **(C)** severity and age (Mann–Whitney test).

Median age on admission was 58 years (Q_1_–Q_3_: 44–70 years, range: 18–95 years) (Figure 1A). Deceased were older than survivors [65 years (Q_1_–Q_3_: 56–78 years) vs. 58 years (Q_1_–Q_3_: 44–70 years), *p* = 0.017, respectively] (Figure 1B). The age difference between severe and non-severe cases was of borderline significance [61 years (Q_1_–Q_3_: 48–71 years) vs. 58 years (Q_1_–Q_3_: 43–70 years), *p* = 0.076] (Figure 1C), as well as the detected weak positive correlation between age and LOH (*r* = 0.055, *p* = 0.058) (Supplementary Appendix 4). Interestingly, patients developing local complications were younger than those not doing so [56 years (Q_1_–Q_3_: 43–68 years) vs. 59 years (Q_1_–Q_3_: 44–71 years), respectively, *p* = 0.028]. The association is true for necrosis (*p* = 0.049) and fluid collections (*p* = 0.095), unlike for pseudocysts (*p* = 0.839) (Supplementary Appendix 5). On the contrary, patients developing systemic complications were older than those not doing so [62 years (Q_1_–Q_3_: 50.5–74 years) vs. 58 years (Q_1_–Q_3_: 43–70 years), respectively, *p* = 0.008]. Specifically, respiratory (*p* = 0.001) and heart failure (*p* = 0.009) were age-dependent (Supplementary Appendix 5).

#### Comorbidities (CCI) Strongly Influences the Outcomes of AP in Univariate Models

**FIGURE 2:**
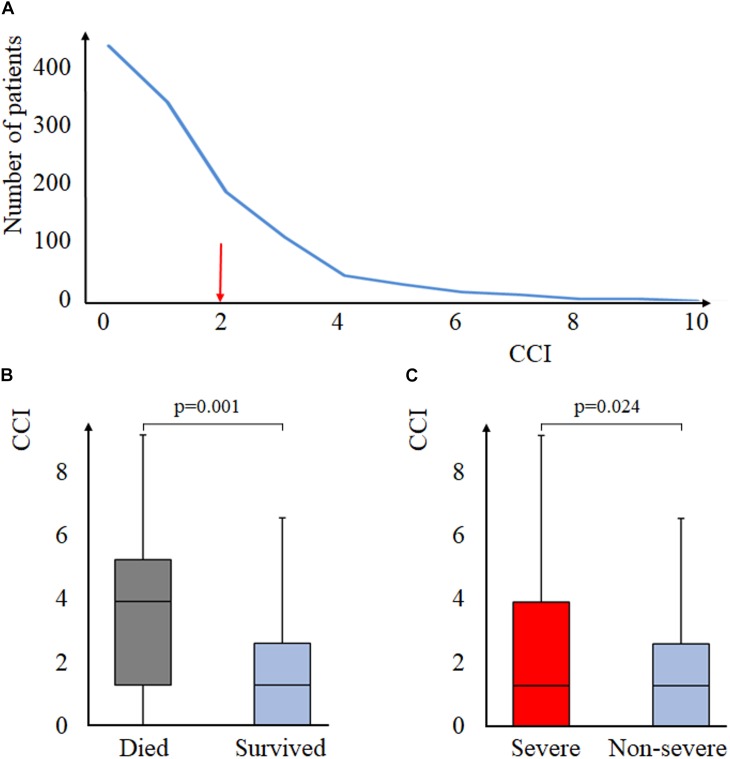
Charlson Comorbidity Score and AP. **(A)** distribution of CCI in the study population, the red arrow indicates the median CCI of the population. **(B)** mortality and CCI (Mann–Whitney test). **(C)** severity and CCI (Mann–Whitney test). CCI, Charlson Comorbidity Index.

Median CCI was 2 (Q_1_–Q_3_: 0–2, range: 0–10) (Figure 2A). Deceased had higher CCI than survivors [3 (Q_1_–Q_3_: 1–4) vs. 1 (Q_1_–Q_3_: 0–2), *p* = 0.001, respectively], as well as those with severe AP [1 (Q_1_–Q_3_: 0–3) vs. 1 (Q_1_–Q_3_: 0–2), *p* = 0.024] compared to those with non-severe AP, respectively (Figure 2B–C). A weak, significant, positive correlation was detected between age and CCI (*r* = 0.073, *p* = 0.012) (Supplementary Appendix 4). Local complications seemed independent of CCI (*p* = 0.259), as were fluid collections (*p* = 0.515), pseudocysts (*p* = 0.456), and necrosis (*p* = 0.558) (Supplementary Appendix 6). Systemic complications were associated with higher CCI (*p* < 0.001). This association applies to respiratory failure (*p* < 0.001), as well (Supplementary Appendix 6).

#### Age Correlates With CCI in a Univariate Model

**FIGURE 3:**
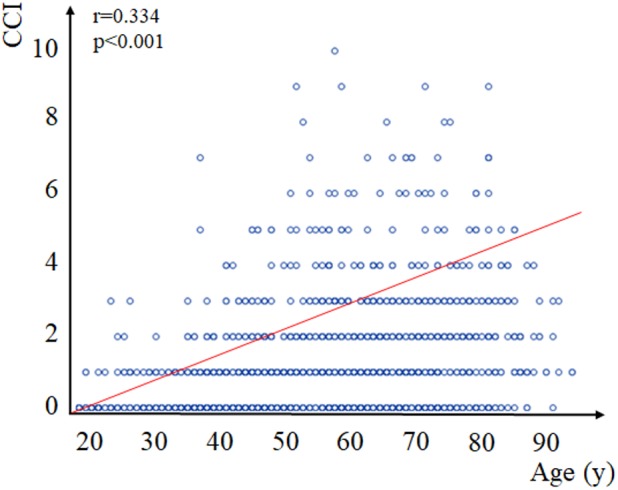
Correlation between age and Charlson Comorbidity Index (CCI). Spearman’s correlation established a significant positive correlation of moderate strength (*r* = 0.334, *p* < 0.001) between age on admission and CCI. CCI, Charlson Comorbidity Index.

We observed a moderate, positive correlation between age and CCI (*r* = 0.334, *p* < 0.001) (Figure 3).

Analyzing the association between the individual comorbidities (i.e., the components of CCI) and age, patients with previous myocardial infarction, co-existing congestive heart failure, peripheral arterial disease, and cerebrovascular disease were significantly older than those without these conditions (*p* < 0.001 for each). These associations applied to chronic pulmonary diseases and dementia (*p* < 0.001 for both), as well as to peptic ulcers/erosions (*p* = 0.015). Both diabetes with and without complications were associated with older age (*p* < 0.001).

Patients with malignant tumors were older (*p* < 0.001) but we failed to detect this association regarding metastatic tumors (*p* = 0.112), probably due to low event rates. The latter may apply to autoimmune diseases (*p* = 0.961).

Interestingly, patients with mild liver disease were younger than their healthy counterparts (*p* < 0.001); however, this difference disappeared regarding moderate and severe liver diseases (*p* = 0.555).

#### Aging and Comorbidities (CCI) Affect the Outcomes of AP Discrepantly in Multivariate Models

Summaries of multivariate analysis are presented in Table 2 and Supplementary Appendix 7, raw data are presented in Supplementary Appendix 8. The exclusive predictor of mortality was a CCI ≥ 3 (ß = 1.50; OR = 4.48; CI: 1.57–12.80); in accordance, the main predictor of severe AP was a CCI ≥ 3 (ß = 0.74; OR = 2.10, CI: 1.08–4.09), though the middle- and old-aged were exposed to a severe episode with a high OR of borderline significance. Unexpectedly, the middle-aged were more likely to spend ≥9 days in hospital. Along with this, the only predictors of local complications (including pancreatic necrosis) was to be middle-aged (ß = 1.17; OR = 3.21, CI: 1.26–8.19). On the contrary, the middle- and old-aged were about eight times more likely to develop systemic complications than their younger counterparts (β = 2.19, OR = 7.82, CI: 1.06–57.79 and β = 2.06, OR = 8.93, CI: 1.20–66.79, respectively), though comorbidities were important determinants, as well.

**Table 2:** Joint effect of aging and comorbidities on the outcomes of AP.

Variables	Deceased vs. survivors			Severe vs. mild AP			LOH ≤9 days vs. LOH >9 days		
	
	β	OR (95% CI)	*p*-value	β	OR (95% CI)	*p*-value	β	OR (95% CI)	*p*-value
**Age categories**									
18–34 years (young-aged)	NA^a^	NA^a^	0.961	0	1 (reference)		0	1 (reference)	
35–64 years (middle-aged)	0.76	0.76 (0.35–1.67)	0.493	2.00	7.40 (0.99–55.31)	0.051	0.62	1.86 (1.22–2.83)	0.004
>65 years (old-aged)	0	1 (ref)		1.93	6.92 (0.91–52.70)	0.062	0.40	1.50 (0.96–2.33)	0.073
**Comorbidity categories**									
CCI = 0 (none)	0	1 (reference)		0	1 (reference)		0	1 (reference)	
CCI = 1 (mild)	0.11	1.12 (0.32–3.90)	0.863	0.04	1.04 (0.52–2.08)	0.911	0.00	1.00 (0.75–1.34)	0.983
CCI = 2 (moderate)	0.09	1.10 (0.26–4.68)	0.900	−0.02	0.98 (0.45–2.24)	0.960	0.30	1.35 (0.95–1.92)	0.092
CCI > 2 (severe)	1.50	4.48 (1.57–12.80)	0.005	0.74	2.10 (1.08–4.09)	0.029	0.15	1.16 (0.83–1.62)	0.387

*Red highlights indicate p < 0.05, orange highlights indicate p < 0.10 but ≥0.05. AP, acute pancreatitis; Charlson Comorbidity Index; CI, confidence interval; LOH, length of hospitalization; NA, not applicable; OR, odds ratio. ^a^analysis is impossible due to zero events.*

#### Individual Comorbidities Are Important Predictors of the Outcomes of AP in Univariate and Multivariate Models

**FIGURE 4:**
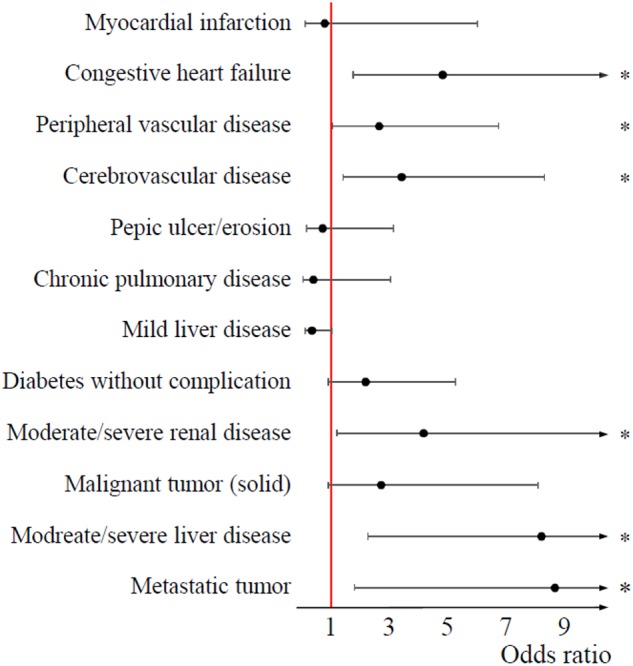
Forest plot on the effect of individual comorbidities on mortality. 95% confidence intervals did not cross the boundary of significance (red, vertical line at an odds ratio of (1) regarding six comorbid conditions: congestive heart failure, peripheral vascular disease, cerebrovascular disease, moderate/severe renal disease, moderate/severe liver disease, and metastatic tumor (asterisks indicate a *p*-value less than 0.05). These comorbidities were associated with higher mortality.

Summaries of univariate and multivariate statistics of individual comorbidities, together with raw data, are presented in Supplementary Appendices 8–10. In univariate analysis, out of the six comorbidities associated with higher mortality, moderate/severe liver diseases and metastatic solid tumors proved to be the strongest predictors (OR = 8.04, CI: 2.22–29.13 and OR = 8.47, CI: 1.78–40.23, respectively) (Figure 4). Peripheral vascular diseases, cerebrovascular diseases, and diabetes without complications predicted severe AP. Patients with mild liver diseases were two times more likely to develop local complications, including necrotizing pancreatitis (OR = 1.86, CI: 1.25–2.75). Congestive heart failure, peripheral vascular diseases, cerebrovascular diseases, chronic pulmonary diseases, and diabetes without complications were associated with a higher rate of systemic complications. Preexisting cardiovascular, renal, and pulmonary diseases predicted the development of respiratory, heart, and renal decompensation, respectively. Interestingly, pre-existing moderate/severe liver diseases and malignant tumors were strongly associated with cardiac decompensation (OR = 7.16, CI: 1.55–33.21 and OR = 4.09, CI: 1.32–12.64, respectively). Multivariate analysis only minimally changed the direction of main associations.

## Discussion

### Summary of Findings

We aimed to clarify whether aging or comorbidities are decisive for determining the outcomes of AP. All the outcomes, except for local complications, proved to be dependent on both age and CCI in univariate analysis. As opposed to this, multivariate analysis revealed that patients suffering from severe comorbidities were about 4.5 times more likely to have a fatal episode of AP and about two times more likely to develop severe AP than those having no comorbidities, whereas age predicted these outcomes with high OR and borderline significance. In contrast, the middle- and old-aged (but not those with severe comorbidities) were more likely to spend at least 9 days in hospital, as compared to their young counterparts. Moreover, aging and comorbidities influenced the development of local and systemic complications in a completely different manner.

Frequency of comorbidities and distribution of age were similar in our cohort of AP cases to that of the large series in the literature (Frey et al., 2007; Singla et al., 2009; Murata et al., 2011, 2015; Akshintala et al., 2013; McNabb-Baltar et al., 2014).

Although mortality of populations is widely reported, studies on the effect of aging yielded controversial results. Some indicated that each year increase in age may result in an OR = 1.01–1.04 (*p* < 0.05) increase in mortality (Singla et al., 2009; Akshintala et al., 2013; McNabb-Baltar et al., 2014); however, the detection of this statistically significant but probably clinically less prominent increment might have been attained due to large sample sizes. High mortality of older age groups is frequently reported (Frey et al., 2007; Murata et al., 2011; Mendez-Bailon et al., 2015), as are the effects of severe comorbidities: they are strong, independent predictors of mortality in AP (Frey et al., 2007; Singla et al., 2009; Murata et al., 2011, 2015; Akshintala et al., 2013; McNabb-Baltar et al., 2014; Mendez-Bailon et al., 2015; Lee et al., 2016), as confirmed by our study, as well. Our results are in line with previous findings in a cohort of patients over 70 years stating that pre-existing cardiovascular, malignant, and renal diseases predicted mortality (Murata et al., 2015).

No studies investigated the effects of comorbidities on AP severity graded by the revised Atlanta criteria (Sarr, 2013). In our study, patients with severe AP were older and had higher CCI than those developing moderate AP. Besides that a CCI ≥ 3 is an independent predictor of severe AP, middle and old age should be considered a strong risk factor in multivariate analysis (including age and CCI categories).

The middle- and old-aged patients were more likely to stay ≥9 day in hospital as compared to younger counterparts. We found no association between LOH and comorbidities, which may oppose previous research (Murata et al., 2011, 2015; Francisco et al., 2013). A possible explanation for this discrepancy may be that we handled LOH as a dichotomous variable in multivariate analysis due to non-normal distribution of data. No studies have analyzed the effect of individual comorbidities on LOH; in our cohort of patients myocardial infarction, mild liver diseases as well as middle and old age predisposed to longer LOH.

Interestingly, patients with local complications and necrosis were younger but do not have higher CCI than those not developing them. Only being middle-aged was an independent predictor of local complications and necrosis. Two small studies reported non-significant associations between comorbidities and local complications (Uomo et al., 1998; Weitz et al., 2016). One study reported on 2-week organ failure, which found that only the number of comorbidities, but not age, was a significant predictor (Frey et al., 2007). On the contrary in our study, the strongest predictor of organ failure was aging: the middle- and old aged were about 8 times more likely to develop organ failure than their younger counterparts, while having severe comorbidities proved to be a weak but significant predictor, as well.

### Strengths and Weaknesses

Our study has several strengths. First of all, this is the first report analyzing the joint effect of aging and comorbidities on AP severity and local complications in a non-selected cohort of AP cases with multivariate statistics. Secondly, manual assessment of CCI by a trained investigator provides a sufficient accuracy and might be superior in homogeneity over claims data (Kieszak et al., 1999) upon which most population-based studies rely. Third, precise data collection and consistent data management of the AP Registry with uniform recording of diagnosis, severity, and complications across centers improve the reliability of data and, therefore, strengthen our conclusions (Sarr, 2013).

However, authors must acknowledge that the study is limited by the number of reasons. Data collected are limited to adult (18–95 years). Despite the high case number, event numbers concerning some outcomes limited the analysis. To overcome this, we merged similar items of CCI (e.g., malignant tumors) when imputing them in multivariate models, as seen in other works (Murata et al., 2015). Distribution of continuous variables proved to be non-normal so that multivariate regression was not performed in terms of LOH. Instead, a dichotomized logistic regression model was used. Similarly, the non-normal distribution of age and CCI forced us to set up age and comorbidity categories in multivariate analysis. Despite the four-level data checking system, imprecision of data recording cannot be excluded.

## Conclusion

**FIGURE 5:**
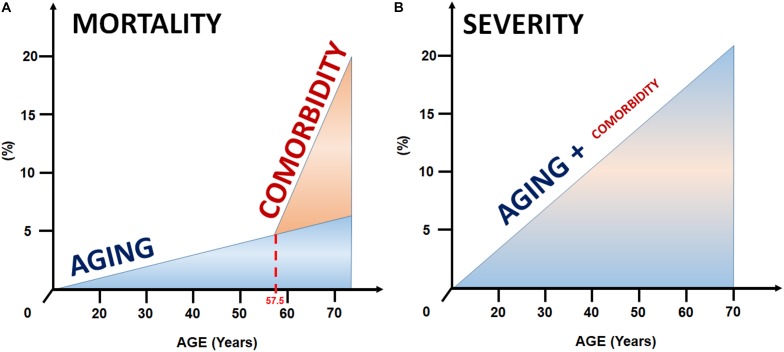
Model for the joint effect of aging and comorbidities on mortality and severity. **(A)** The excess in mortality in the elderly is likely to be explained by the increment in comorbidities with aging. **(B)** In contrast, age seems to be the strongest predictor of the severity of AP, whereas comorbidities have a less prominent effect.

Our results confirm that both aging and comorbidities modify the outcomes of AP, however, discrepantly. The increment in mortality associated with an older age in the meta-analysis of Marta et al. might be explained by the additive effects of comorbidities (Figure 5). Taken together, these results support that CCI, together with age, should be incorporated into the predictive scores in AP to increase the accuracy of prediction. Studies validating the implementation of CCI-based predictive scores are awaited.

## Author’s Note

There is a Part I of this publication in which a metaanalysis of 194 702 cases showed that additional factors play a crucial role in mortality of acute pancreatitis above 59 years of age (Figures 7, 11 – https://www.frontiersin.org/articles/10.3389/fphys.2019.00328/full; doi: 10.3389/fphys.2019.00328). The results of this article proved that mortality of acute pancreatitis is rather determined by the presence of comorbid conditions (Figure 5 and Table 2).

## Author Contributions

ZS, PH, and ÁV contributed to the design of the research. GK, EF, DD, BK, KM, KK, IS, IT, LG, MP, PS, SG, MV, JH, and TT performed the data collection. AS coordinated data collection and controlled data quality. ZS assessed the comorbidities and calculated comorbidity scores. NG and ZS processed the data, performed the analysis, and drafted the manuscript. ZS and AP designed the figures. DP and FI critically revised the manuscript. PH supervised and coordinated the work. All authors discussed the results and commented on the manuscript.

## Conflict of Interest Statement

The authors declare that the research was conducted in the absence of any commercial or financial relationships that could be construed as a potential conflict of interest.

## Supplementary Material

APPENDIX 1 Tables of demography and representativeness of the study population. Distribution of centers recruiting the study population.

APPENDIX 2 Data quality.

APPENDIX 3 Figures of demography and representativeness of the study population.

APPENDIX 4 Correlation between age and LOH (A panel), and CCI and LOH (B panel).

APPENDIX 5 Aging and complications in acute pancreatitis.

APPENDIX 6 Comorbidities and complications in acute pancreatitis.

APPENDIX 7 Joint effect of aging and comorbidities on local and systemic complications of AP.

APPENDIX 8 Data used in multivariate analysis.

APPENDIX 9 Results of univariate analysis on the effects of individual comorbidities on the outcomes of acute pancreatitis.

APPENDIX 10 Results of multivariate analysis on the effects of individual comorbidities on the outcomes of acute pancreatitis.

## Abbreviations

AP: acute pancreatitis
APACHE: Acute Physiology and Chronic Health Evaluation
BISAP: Bedside Index of Severity in Acute Pancreatitis
CCI: Charlson Comorbidity Index
CI: confidence interval
LOH: length of hospitalization
OR: odds ratio.

## References

[B1] Akshintala V. S. Hutfless S. M. Yadav D. Khashab M. A. Lennon A. M. Makary M. A. (2013). A population-based study of severity in patients with acute on chronic pancreatitis. *Pancreas* 42 1245–1250. 10.1097/MPA.0b013e3182a85af3 24152950

[B2] Alsamarrai A. Das S. L. Windsor J. A. Petrov M. S. (2014). Factors that affect risk for pancreatic disease in the general population: a systematic review and meta-analysis of prospective cohort studies. *Clin. Gastroenterol. Hepatol.* 12 1635.e5–1644.e5. 10.1016/j.cgh.2014.01.038 24509242

[B3] Charlson M. E. Pompei P. Ales K. L. Mackenzie C. R. (1987). A new method of classifying prognostic comorbidity in longitudinal studies: development and validation. *J. Chronic Dis.* 40 373–383. 10.1016/0021-9681(87)90171-8 3558716

[B4] Fagenholz P. J. Fernandez-Del Castillo C. Harris N. S. Pelletier A. J. Camargo C. A. Jr. (2007). Direct medical costs of acute pancreatitis hospitalizations in the United States. *Pancreas* 35 302–307. 10.1097/MPA.0b013e3180cac24b 18090234

[B5] Fan S. T. Choi T. K. Lai C. S. Wong J. (1988). Influence of age on the mortality from acute pancreatitis. *Br. J. Surg.* 75 463–466. 10.1002/bjs.1800750520 3390679

[B6] Francisco M. Valentin F. Cubiella J. Fernandez-Seara J. (2013). Factors related to length of hospital admission in mild interstitial acute pancreatitis. *Rev. Esp. Enferm. Dig.* 105 84–92. 10.4321/S1130-01082013000200005 23659507

[B7] Frenkel W. J. Jongerius E. J. Mandjes-Van Uitert M. J. Van Munster B. C. De Rooij S. E. (2014). Validation of the Charlson comorbidity index in acutely hospitalized elderly adults: a prospective cohort study. *J. Am. Geriatr. Soc.* 62 342–346. 10.1111/jgs.12635 24521366

[B8] Frey C. Zhou H. Harvey D. White R. H. (2007). Co-morbidity is a strong predictor of early death and multi-organ system failure among patients with acute pancreatitis. *J. Gastrointest. Surg.* 11 733–742. 10.1007/s11605-007-0164-5 17417710

[B9] Hamada S. Masamune A. Kikuta K. Hirota M. Tsuji I. Shimosegawa T. (2014). Nationwide epidemiological survey of acute pancreatitis in Japan. *Pancreas* 43 1244–1248. 10.1097/MPA.0000000000000200 25084001

[B10] Kieszak S. M. Flanders W. D. Kosinski A. S. Shipp C. C. Karp H. (1999). A comparison of the Charlson comorbidity index derived from medical record data and administrative billing data. *J. Clin. Epidemiol.* 52 137–142. 10.1016/S0895-4356(98)00154-1 10201654

[B11] Larvin M. McMahon M. J. (1989). APACHE-II score for assessment and monitoring of acute pancreatitis. *Lancet* 2 201–205. 10.1016/S0140-6736(89)90381-4 2568529

[B12] Lee P. J. Bhatt A. Lopez R. Stevens T. (2016). Thirty-day readmission predicts 1-Year mortality in acute pancreatitis. *Pancreas* 45 561–564. 10.1097/MPA.0000000000000463 26390423

[B13] Marventano S. Grosso G. Mistretta A. Bogusz-Czerniewicz M. Ferranti R. Nolfo F. (2014). Evaluation of four comorbidity indices and Charlson comorbidity index adjustment for colorectal cancer patients. *Int. J. Colorectal. Dis.* 29 1159–1169. 10.1007/s00384-014-1972-1 25064390

[B14] McNabb-Baltar J. Ravi P. Isabwe G. A. Suleiman S. L. Yaghoobi M. Trinh Q. D. (2014). A population-based assessment of the burden of acute pancreatitis in the United States. *Pancreas* 43 687–691. 10.1097/MPA.0000000000000123 24694835

[B15] Mendez-Bailon M. De Miguel Yanes J. M. Jimenez-Garcia R. Hernandez-Barrera V. Perez-Farinos N. Lopez-De-Andres A. (2015). National trends in incidence and outcomes of acute pancreatitis among type 2 diabetics and non-diabetics in Spain (2001-2011). *Pancreatology* 15 64–70. 10.1016/j.pan.2014.11.004 25500341

[B16] Murata A. Matsuda S. Mayumi T. Okamoto K. Kuwabara K. Ichimiya Y. (2012). Multivariate analysis of factors influencing medical costs of acute pancreatitis hospitalizations based on a national administrative database. *Dig. Liver Dis.* 44 143–148. 10.1016/j.dld.2011.08.011 21930445

[B17] Murata A. Matsuda S. Mayumi T. Yokoe M. Kuwabara K. Ichimiya Y. (2011). Effect of hospital volume on clinical outcome in patients with acute pancreatitis, based on a national administrative database. *Pancreas* 40 1018–1023. 10.1097/MPA.0b013e31821bd233 21926541

[B18] Murata A. Ohtani M. Muramatsu K. Matsuda S. (2015). Influence of comorbidity on outcomes of older patients with acute pancreatitis based on a national administrative database. *Hepatobiliary Pancreat. Dis. Int.* 14 422–428. 10.1016/S1499-3872(15)60398-8 26256088

[B19] Ng A. C. Chow V. Yong A. S. Chung T. Kritharides L. (2013). Prognostic impact of the Charlson comorbidity index on mortality following acute pulmonary embolism. *Respiration* 85 408–416. 10.1159/000342024 23147354

[B20] Parniczky A. Kui B. Szentesi A. Balazs A. Szucs A. Mosztbacher D. (2016). Prospective, multicentre, nationwide clinical data from 600 cases of acute pancreatitis. *PLoS One* 11:10. 10.1371/journal.pone.0165309 27798670 PMC5087847

[B21] Quan H. Sundararajan V. Halfon P. Fong A. Burnand B. Luthi J. C. (2005). Coding algorithms for defining comorbidities in ICD-9-CM and ICD-10 administrative data. *Med. Care* 43 1130–1139. 10.1097/01.mlr.0000182534.19832.83 16224307

[B22] Ranson J. H. Rifkind K. M. Roses D. F. Fink S. D. Eng K. Localio S. A. (1974). Objective early identification of severe acute pancreatitis. *Am. J. Gastroenterol.* 61 443–451. 4835417

[B23] Roberts S. E. Akbari A. Thorne K. Atkinson M. Evans P. A. (2013). The incidence of acute pancreatitis: impact of social deprivation, alcohol consumption, seasonal and demographic factors. *Aliment. Pharmacol. Ther.* 38 539–548. 10.1111/apt.12408 23859492 PMC4489350

[B24] Samokhvalov A. V. Rehm J. Roerecke M. (2015). Alcohol consumption as a risk factor for acute and chronic pancreatitis: a systematic review and a series of meta-analyses. *EBioMedicine* 2 1996–2002. 10.1016/j.ebiom.2015.11.023 26844279 PMC4703772

[B25] Sarr M. G. (2013). 2012 revision of the Atlanta classification of acute pancreatitis. *Pol. Arch. Med. Wewn.* 123 118–124. 10.20452/pamw.1627 23396317

[B26] Singla A. Csikesz N. G. Simons J. P. Li Y. F. Ng S. C. Tseng J. F. (2009). National hospital volume in acute pancreatitis: analysis of the Nationwide Inpatient Sample 1998-2006. *HPB* 11 391–397. 10.1111/j.1477-2574.2009.00072.x 19768143 PMC2742608

[B27] Spanier B. Bruno M. J. Dijkgraaf M. G. (2013). Incidence and mortality of acute and chronic pancreatitis in the Netherlands: a nationwide record-linked cohort study for the years 1995-2005. *World. J. Gastroenterol.* 19 3018–3026. 10.3748/wjg.v19.i20.3018 23716981 PMC3662941

[B28] Uomo G. Talamini G. Rabitti P. G. Cataldi F. Cavallera A. Rengo F. (1998). Influence of advanced age and related comorbidity on the course and outcome of acute pancreatitis. *Ital. J. Gastroenterol. Hepatol.* 30 616–621. 10076785

[B29] Vasilopoulos T. Kotwal A. Huisingh-Scheetz M. J. Waite L. J. Mcclintock M. K. Dale W. (2014). Comorbidity and chronic conditions in the National Social Life, Health and Aging Project (NSHAP), Wave 2. *J. Gerontol. B Psychol. Sci. Soc. Sci.* 69(Suppl. 2), S154–S165. 10.1093/geronb/gbu025 25360017 PMC4303089

[B30] Weitz G. Woitalla J. Wellhoner P. Schmidt K. J. Buning J. Fellermann K. (2016). Comorbidity in acute pancreatitis relates to organ failure but not to local complications. *Z. Gastroenterol.* 54 226–230. 10.1055/s-0041-106593 27043885

[B31] Working Group Iap/Apa Acute Pancreatitis Guidelines. (2013). IAP/APA evidence-based guidelines for the management of acute pancreatitis. *Pancreatology* 13(4 Suppl. 2), e1–e15. 10.1016/j.pan.2013.07.063 24054878

[B32] Wu B. U. Johannes R. S. Sun X. Tabak Y. Conwell D. L. Banks P. A. (2008). The early prediction of mortality in acute pancreatitis: a large population-based study. *Gut* 57 1698–1703. 10.1136/gut.2008.152702 18519429

[B33] Yadav D. Lowenfels A. B. (2013). The epidemiology of pancreatitis and pancreatic cancer. *Gastroenterology* 144 1252–1261. 10.1053/j.gastro.2013.01.068 23622135 PMC3662544

[B34] Yadav D. Ng B. Saul M. Kennard E. D. (2011). Relationship of serum pancreatic enzyme testing trends with the diagnosis of acute pancreatitis. *Pancreas* 40 383–389. 10.1097/MPA.0b013e3182062970 21283039

