# Supplementary material for: Aging and Comorbidities in Acute Pancreatitis II.: A Cohort-Analysis of 1203 Prospectively Collected Cases
Source: Front Physiol. 2019 Apr 2;9:1776. doi: 10.3389/fphys.2018.01776 (PMC6454835; doi:10.3389/fphys.2018.01776)
Supplement: APPENDIX 1 — Tables of demography and representativeness of the study population. Distribution of centers recruiting the study population. [file Data_Sheet_1.PDF]

Supplementary Appendix 1. Tables of demography and representativeness of the study population. Distribution of centres recruiting the study population

Demography of study population including 1203 cases compared to that of AP Registry including 1241 cases.

	AP Registry (n=1241)	Study population* (n=1203)
Age, median (Q_1-Q_3)	58 (44-70)	58 (44-70)
Sex, n_{male} ($\%_{male}$)	695 (56.0)	670 (55.7)
Etiology (pure)		
Biliary, n (%)	536 (43.2)	528 (43.9)
Alcoholic, n (%)	284 (22.9)	269 (22.4)
Hypertriglyceridemic, n (%)	70 (5.6)	69 (5.7)
Mortality, n (%)	29 (2.3)	28 (2.3)
Severity of pancreatitis		
Mild, n (%)	852 (68.7)	825 (68.6)
Moderate, n (%)	320 (25.8)	313 (26.0)
Severe, n (%)	69 (5.6)	65 (5.4)
Length of hospitalization, median (Q_1-Q_3)	9 (6-14)	9 (7-14)
Local complications, n (%)	367 (29.6)	358 (29.8)
Fluid collection, n (%)	309 (24.9)	303 (25.2)
Pseudocyst, n (%)	123 (9.9)	120 (10.0)
Necrosis, n (%)	114 (9.2)	111 (9.2)
Systemic complications, n (%)	96 (7.7)	92 (7.7)
Respiratory failure, n (%)	57 (4.6)	55 (4.6)
Heart failure, n (%)	19 (1.5)	19 (1.6)
Renal failure, n (%)	34 (2.7)	33 (2.7)
Charlson Comorbidity Index, median (Q_1-Q_3)	-	2 (0-2)
Severity of comorbidities		
No comorbidities, n (%)	-	444 (36.9)
Mild comorbidities, n (%)	-	345 (28.7)
Moderate comorbidities, n (%)	-	190 (15.8)
Severe comorbidities, n (%)	-	224 (18.6)

Continuous variables are presented in median with quartiles (Q_1-Q_3), categorical variables are presented in frequencies (n) with percentages of total (%). *Study population is representative of AP Registry in terms of all variables listed ($p>0.05$).

Distribution of Hungarian centers recruiting 1203 cases of acute pancreatitis

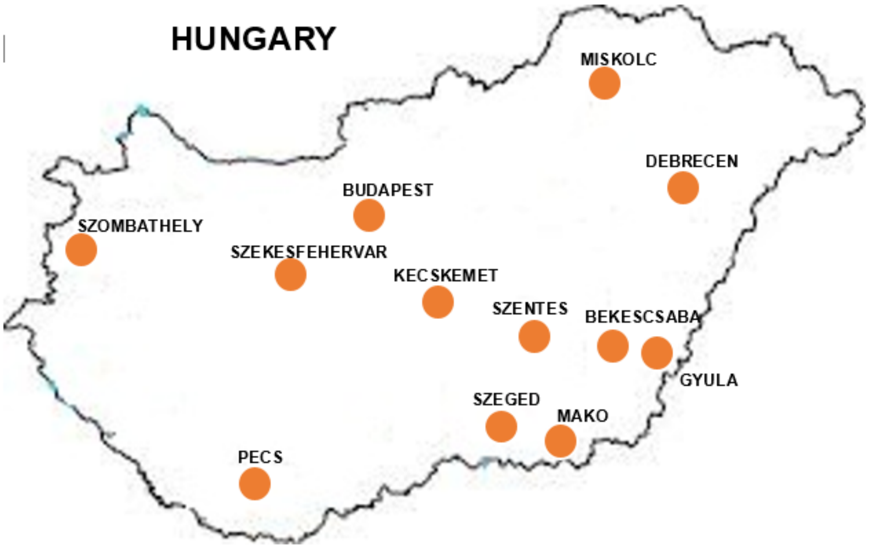

City	Center	N^0 of patients
**Budapest**	Bajcsy-Zsilinszky Hospital	137
	Joint Saint Istvan and Saint Laszlo Hospitals	4
	Heim Pál National Institute of Pediatrics	1
**Békéscsaba**	Dr. Réthy Pál Hospital	53
**Debrecen**	Second Department of Medicine, University of Debrecen	76
	Institute of Surgery, University of Debrecen	7
**Gyula**	Pándy Kálmán Hospital of County Békés	26
**Kecskemét**	Bács-Kiskun County Hospital	11
**Makó**	Healthcare Center of County Csongrád	10
**Miskolc**	Borsod-Abaúj-Zemplén County Hospital and University Teaching Hospital	11
**Pécs**	First Department of Medicine, University of Pecs	360
**Szeged**	First Department of Medicine, University of Szeged	198
	Second Department of Medicine, University of Szeged	74
	Emergency Unit, University of Szeged	10
	Department of Surgery, University of Szeged	4
**Szentes**	Department of Gastroenterology, Dr. Bugyi István Hospital	10
**Szombathely**	Markusovszky University Teaching Hospital	8
**Székesfehérvár**	Szent György University Teaching Hospital of Fejér County	199
	**Total N^0 of patients**	**1203**
