# Supplementary material for: Aging and Comorbidities in Acute Pancreatitis II.: A Cohort-Analysis of 1203 Prospectively Collected Cases
Source: Front Physiol. 2019 Apr 2;9:1776. doi: 10.3389/fphys.2018.01776 (PMC6454835; doi:10.3389/fphys.2018.01776)
Supplement: APPENDIX 2 — Data quality. [file Data_Sheet_2.PDF]

Supplementary Appendix 2. Data quality of variables in study population including 1203 cases.

Variable	Data quality (%)
Age at the time of admission	100
Sex	100
Etiology	100
Mortality	100
Severity of pancreatitis	100
Length of hospitalization	100
Charlson Comorbidity Index	100
Local complications	99.5
Fluid collections	99.5
Pseudocyst	99.6
Necrosis	99.6
Systemic complications	99.3
Respiratory failure	99.2
Heart failure	99.3
Renal failure	99.3
**Overall data quality**	**99.7**
