# Supplementary material for: Aging and Comorbidities in Acute Pancreatitis II.: A Cohort-Analysis of 1203 Prospectively Collected Cases
Source: Front Physiol. 2019 Apr 2;9:1776. doi: 10.3389/fphys.2018.01776 (PMC6454835; doi:10.3389/fphys.2018.01776)
Supplement: APPENDIX 3 — Figures of demography and representativeness of the study population. [file Data_Sheet_3.PDF]

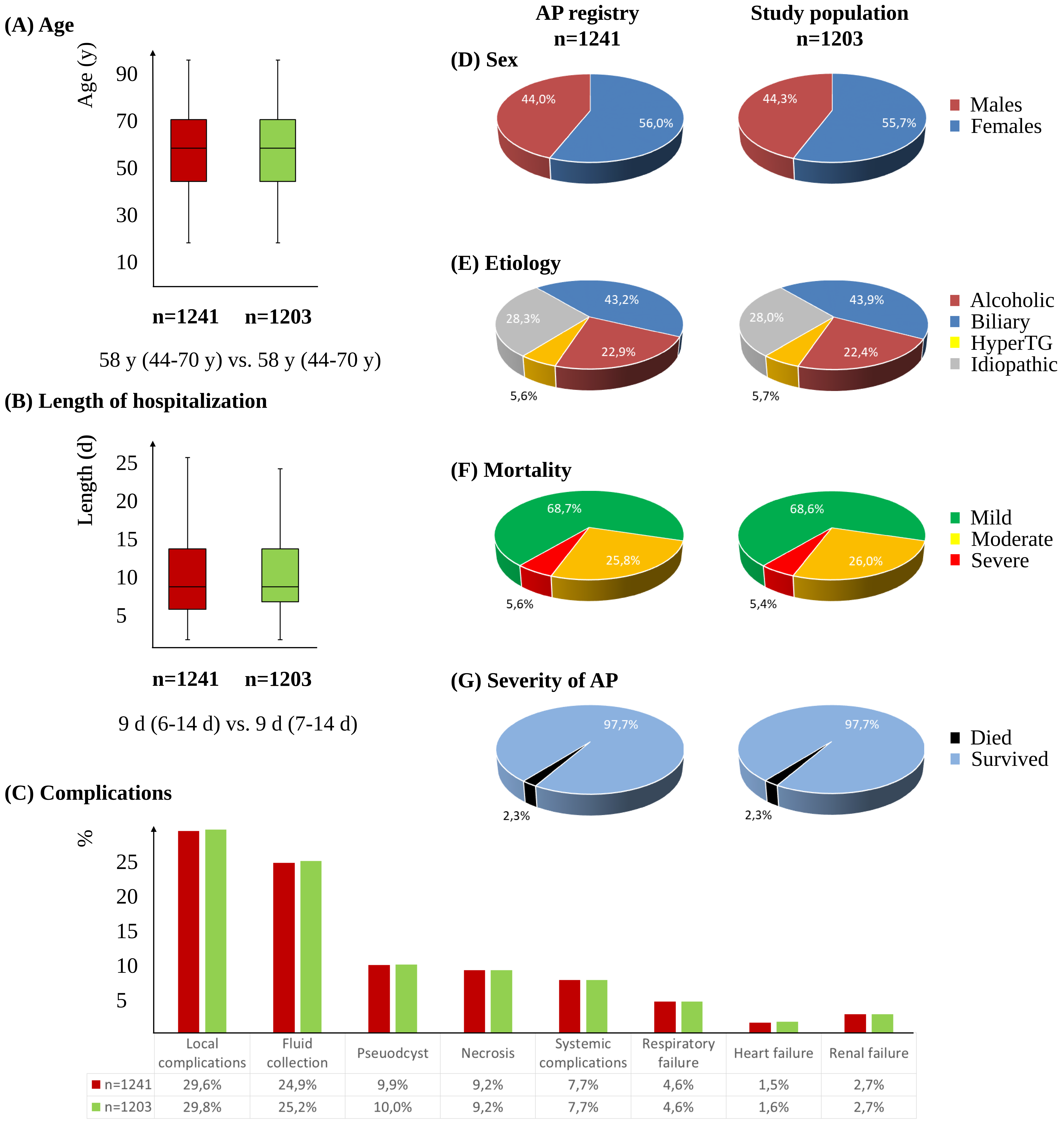

Supporting Appendix 3. Demography and representativeness. Analysis of representativity showed no difference between the features of the population in AP Regsitry (n=1241) and that included in Study Population (n=1203), p≥0.05 for all comparisons. Representativeness of the included population was tested by binomial (sex, etiology, mortality, and complications), one sample median (age and length of hospitalizaton), and Goodness-of-fit χ2 tests (severity of AP).
