# Supplementary material for: Aging and Comorbidities in Acute Pancreatitis II.: A Cohort-Analysis of 1203 Prospectively Collected Cases
Source: Front Physiol. 2019 Apr 2;9:1776. doi: 10.3389/fphys.2018.01776 (PMC6454835; doi:10.3389/fphys.2018.01776)
Supplement: APPENDIX 4 — Correlation between age and LOH (A panel), and CCI and LOH (B panel). [file Data_Sheet_4.PDF]

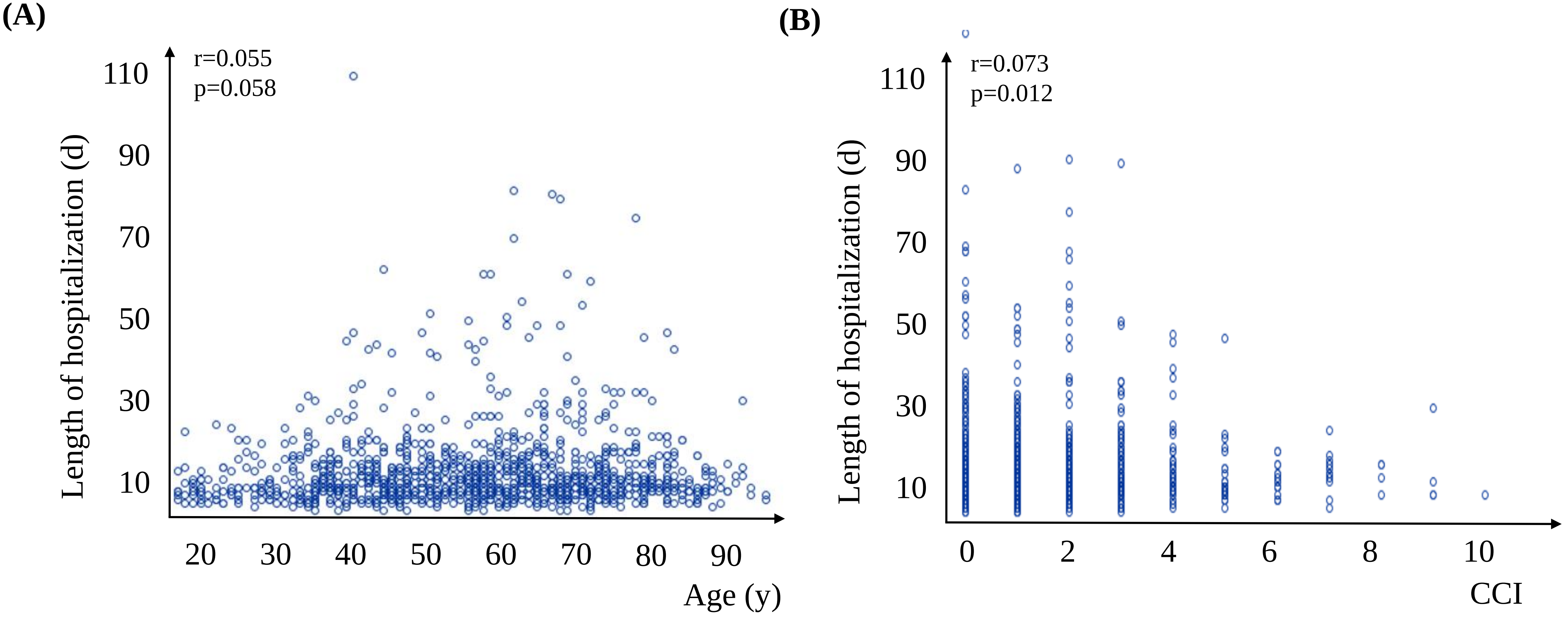

Supplementary Appendix 4. (A) Correlation between age and LOH and (B) CCI and LOH (Spearman's correlation)
