# Supplementary material for: Aging and Comorbidities in Acute Pancreatitis II.: A Cohort-Analysis of 1203 Prospectively Collected Cases
Source: Front Physiol. 2019 Apr 2;9:1776. doi: 10.3389/fphys.2018.01776 (PMC6454835; doi:10.3389/fphys.2018.01776)
Supplement: APPENDIX 5 — Aging and complications in acute pancreatitis. [file Data_Sheet_5.PDF]

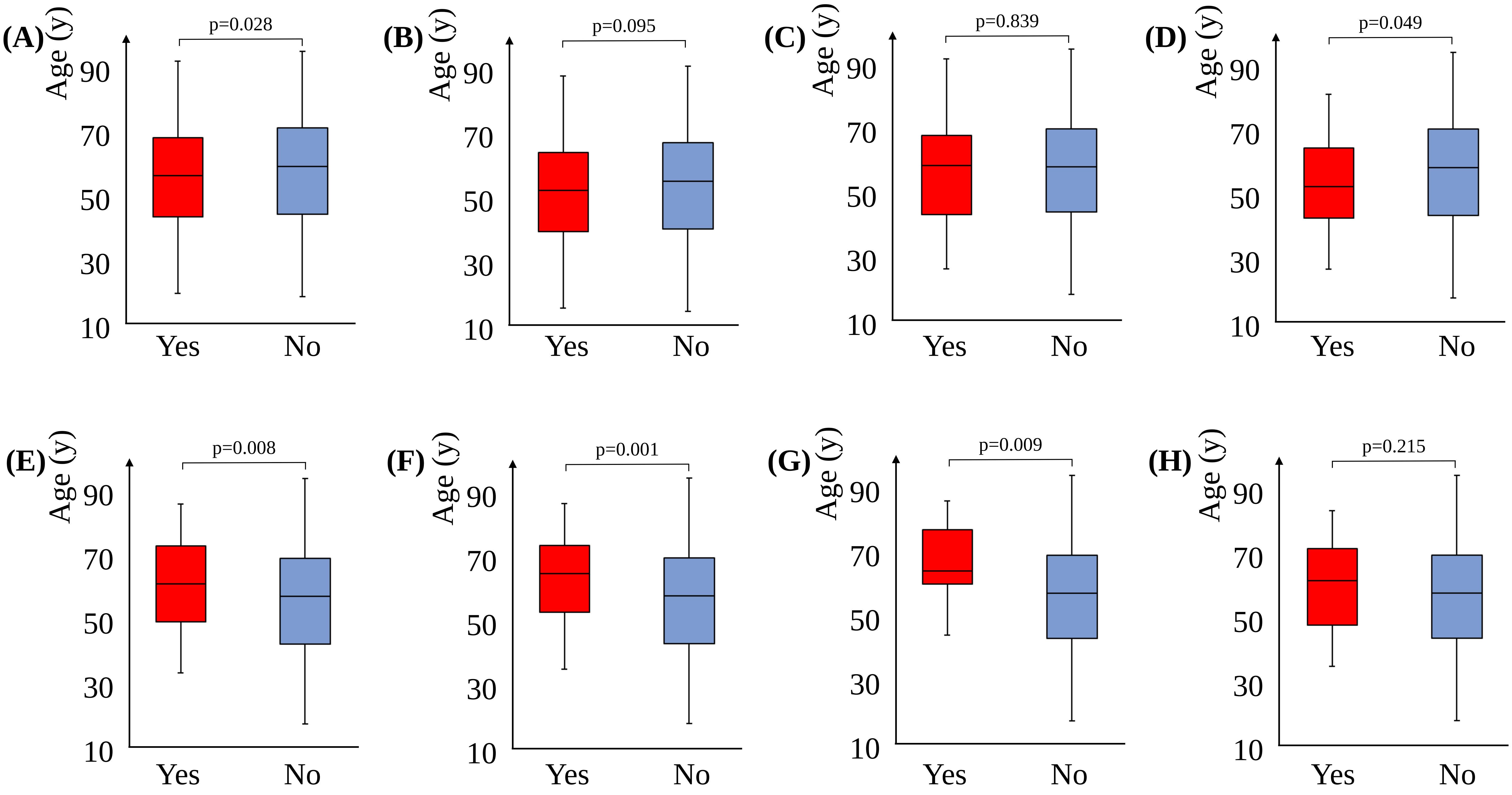

Table 1

		Total n, (%)	Age median (IQR)	p-value
**Local complications** *(Figure A)*	Yes	358 (29.9)	56 (43-68)	0.028*
	No	839 (70.1)	59 (44-71)	
**Fluid collection** *(Figure B)*	Yes	303 (25.3)	56 (43-68)	0.095
	No	894 (74.7)	59 (44-71)	
**Pseudocyst** *(Figure C)*	Yes	120 (10.0)	58.5 (43.5-68)	0.839
	No	1078 (90.0)	58 (44-70)	
**Necrosis** *(Figure D)*	Yes	111 (9.3)	53 (43-65)	0.049*
	No	1087 (90.7)	59 (44-71)	
**Systemic complications** *(Figure E)*	Yes	92 (7.7)	62 (50.5-74)	0.008*
	No	1103 (92.3)	58 (43-70)	
**Respiratory failure** *(Figure F)*	Yes	55 (4.6)	65 (53-74)	0.001*
	No	1139 (95.4)	58 (43-70)	
**Heart failure** *(Figure G)*	Yes	19 (1.6)	65 (61-78)	0.009*
	No	1176 (98.4)	58 (44-70)	
**Renal failure** *(Figure H)*	Yes	33 (2.8)	62 (48-72)	0.215
	No	1162 (97.2)	58 (44-70)	

Supplementary Appendix 5. Aging and complications in acute pancreatitis. (A) any local complication. **(B)** pancreatic fluid collection. **(C)** psudeocyst. **(D)** pancreatic necrosis. **(E)** any systemic complication. **(F)** respiratory failure. **(G)** heart failure. **(H)** renal failure. Groups were compared with Mann-Whitney test. **Table 1** shows the data which the figures rely on. *represents a significant difference between groups.
