# Supplementary material for: Aging and Comorbidities in Acute Pancreatitis II.: A Cohort-Analysis of 1203 Prospectively Collected Cases
Source: Front Physiol. 2019 Apr 2;9:1776. doi: 10.3389/fphys.2018.01776 (PMC6454835; doi:10.3389/fphys.2018.01776)
Supplement: APPENDIX 7 — Joint effect of aging and comorbidities on local and systemic complications of AP. [file Data_Sheet_7.PDF]

Supplementary Appendix 7. Joint effect of aging and comorbidities on local and systemic complications of AP

	Local complications[a]			Fluid collection[a]			Pseudocyst[b]			Necrosis[b]		
	β	OR (95% CI)	p-value	β	OR (95% CI)	p-value	β	OR (95% CI)	p-value	β	OR (95% CI)	p-value
Age categories												
18-34 y (young adults)	0	1 (ref)		0	1 (ref)		0	1 (ref)		0	1 (ref)	
35-64 y (middle-aged adults)	0.75	2.127 (1.300-3.480)*	0.003*	0.63	1.874 (1.124-3.124)*	0.016*	1.20	3.331 (1.305-8.504)*	0.012*	1.17	3.209 (1.257-8.191)*	0.015*
> 65 y (old adults)	0.29	1.332 (0.790-2.244)	0.282	0.23	1.258 (0.731-2.164)	0.407	0.91	2.486 (0.938-6.590)	0.067#	0.53	1.707 (0.633-4.605)	0.291
Comorbidity categories												
CCI=0 (none)	0	1 (ref)		0	1 (ref)		0	1 (ref)		0	1 (ref)	
CCI=1 (mild)	0.20	1.226 (0.895-1.678)	0.204	0.03	1.033 (0.742-1.439)	0.847	-0.24	0.787 (0.489-1.267)	0.325	0.15	1.163 (0.722-1.875)	0.534
CCI=2 (moderate)	0.11	1.116 (0.758-1.644)	0.579	0.05	1.056 (0.705-1.582)	0.791	-0.11	0.894 (0.510-1.569)	0.696	-0.03	0.973 (0.531-1.785)	0.930
CCI>2 (severe)	0.24	1.267 (0.881-1.823)	0.202	0.16	1.169 (0.800-1.709)	0.420	-0.30	0.741 (0.423-1.298)	0.295	-0.20	0.819 (0.447-1.501)	0.519

	Systemic complications[c]			Respiratory failure[d]			Heart failure[c]			Renal failure[c]		
	β	OR (95% CI)	p-value	β	OR (95% CI)	p-value	β	OR (95% CI)	p-value	β	OR (95% CI)	p-value
Age categories												
18-34 y (young adults)	0	1 (ref)		NA[a]	NA[a]	0.964	NA[a]	NA[a]	0.967	NA[a]	NA[a]	0.972
35-64 y (middle-aged adults)	2.19	7.824 (1.059-57.788)*	0.033*	-0.37	0.688 (0.395-1.197)	0.186	-0.31	0.731 (0.289-1.847)	0.508	-0.09	0.919 (0.450-1.875)	0.815
> 65 y (old adults)	2.06	8.933 (1.195-66.794)*	0.044*	0	1 (ref)		0	1 (ref)		0	1 (ref)	
Comorbidity categories												
CCI=0 (none)	0	1 (ref)		0	1 (ref)		0	1 (ref)		0	1 (ref)	
CCI=1 (mild)	0.20	1.217 (0.664-2.232)	0.525	0.02	1.023 (0.444-2.354)	0.958	-0.18	0.835 (0.185-3.765)	0.814	0.88	0.877 (0.323-2.386)	0.797
CCI=2 (moderate)	0.37	1.452 (0.738-2.856)	0.280	0.63	1.870 (0.818-4.275)	0.138	0.32	1.378 (0.302-6.278)	0.679	0.42	0. 416 (0.089-1.957)	0.267
CCI>2 (severe)	0.92	2.505 (1.396-4.494)*	0.002*	0.98	2.658 (1.260-5.605)*	0.010*	1.26	3.522 (1.054-11.770)*	0.041*	2.78	2.779 (1.179-6.551)*	0.020*

*p<0.05, #p<0.10 but ≥0.05. CCI: Charlson Comorbidity Index, [a]not applicable due to zero event number, [a]n=1197, [b]n=1998, [c]n=1995, [d]n=1994, CI: confidence interval, OR: odds ratio.
