# Supplementary material for: Aging and Comorbidities in Acute Pancreatitis II.: A Cohort-Analysis of 1203 Prospectively Collected Cases
Source: Front Physiol. 2019 Apr 2;9:1776. doi: 10.3389/fphys.2018.01776 (PMC6454835; doi:10.3389/fphys.2018.01776)
Supplement: APPENDIX 8 — Data used in multivariate analysis. [file Data_Sheet_8.PDF]

Supplementary Appendix 8. Data used in multivariate analysis

Variables	Mortality n (%)	Severe AP n (%)	LOH ≥9 d n (%)	Local complication n (%)	Fluid collection n (%)	Pseudocyst n (%)	Necrosis n (%)	Systemic complication n (%)	Respiratory failure n (%)	Heart failure n (%)	Renal failure n (%)
Age categories											
18-34 y (young adults)	0 (0.0)	1 (0.8)	40 (33.3)	23 (19.2)	21 (17.5)	5 (4.2)	5 (4.2)	1 (0.8)	0 (0.0)	0 (0.0)	0 (0.0)
35-64 y (middle-aged adults)	13 (2.0)	36 (5.6)	320 (49.7)	223 (34.6)	186 (28.9)	76 (11.8)	77 (12.0)	49 (7.6)	26 (4.0)	9 (1.4)	18 (2.8)
> 65 y (old adults)	14 (3.2)	28 (6.4)	198 (45.1)	112 (25.5)	96 (21.9)	39 (8.9)	29 (6.6)	42 (9.6)	29 (6.6)	10 (2.3)	15 (3.4)
Comorbidity categories											
CCI=0 (none)	5 (1.1)	19 (4.3)	194 (43.7)	120 (27.0)	107 (24.1)	48 (10.8)	40 (9.0)	22 (5.0)	12 (2.7)	4 (0.9)	2 (0.5)
CCI=1 (mild)	5 (1.5)	16 (4.6)	155 (44.9)	110 (31.9)	87 (25.2)	32 (9.3)	37 (10.7)	23 (6.7)	11 (3.2)	3 (0.9)	2 (0.6)
CCI=2 (moderate)	3 (1.6)	9 (4.7)	100 (52.6)	56 (29.5)	48 (25.3)	20 (10.5)	17 (9.0)	16 (8.4)	12 (6.3)	3 (1.6)	2 (1.1)
CCI>2 (severe)	14 (6.3)	21 (9.4)	109 (48.7)	72 (32.1)	61 (27.2)	20 (8.9)	17 (7.6)	31 (13.8)	20 (8.9)	9 (4.0)	2 (0.9)

AP: acute pancreatitis, CCI: Charlson Comorbidity Index, LOH: length of hospitalization
