# Supplementary material for: Aging and Comorbidities in Acute Pancreatitis II.: A Cohort-Analysis of 1203 Prospectively Collected Cases
Source: Front Physiol. 2019 Apr 2;9:1776. doi: 10.3389/fphys.2018.01776 (PMC6454835; doi:10.3389/fphys.2018.01776)
Supplement: APPENDIX 9 — Results of univariate analysis on the effects of individual comorbidities on the outcomes of acute pancreatitis. [file Data_Sheet_9.PDF]

Supplementary Appendix 9.

Comorbidity		Mortality: Total n	Mortality: Died n (%)	Mortality: OR (95% CI)	Severe AP: Total, n	Severe AP: Severe AP 65 (5.4)	Severe AP: OR (95% CI)	Length of hospitalization: Total, n	Length of hospitalization: ≥9 days n (%)	Length of hospitalization: OR (95% CI)
Myocardial infarction	Yes	56	1 (1.8)	0.78 (0.11-5.88)	56	6 (10.7)	2.21 (0.91-5.37)	56	37 (66.1)	2.34 (1.33-4.12)*
	No	1147	26 (2.3)		1147	59 (5.1)		1147	521 (45.4)	
Congestive heart failure	Yes	59	5 (8.5)	4.72 (1.72-12.95)*	59	6 (10.2)	2.08 (0.86-5.04)	59	32 (54.2)	1.39 (0.82-2.35)
	No	1144	22 (1.9)		1144	59 (5.2)		1144	526 (46.0)	
Peripheral vascular disease	Yes	122	6 (4.9)	2.61 (1.03-6.60)*	122	12 (9.8)	2.12 (1.10-4.08)*	122	60 (49.2)	1.13 (0.78-1.65)
	No	1081	21 (1.9)		1081	53 (4.9)		1081	498 (46.1)	
Cerebrovascular disease	Yes	118	7 (5.9)	3.36 (1.39-8.12)*	118	12 (10.2)	2.20 (1.14-2.26) *	118	58 (49.1)	1.13 (0.77-1.65)
	No	1085	20 (1.8)		1085	53 (4.9)		1085	500 (46.1)	
Dementia	Yes	17	0 (0.0)	NA#	17	2 (11.8)	2.38 (0.53-10.62)	17	5 (29.4)	0.48 (0.17-1.36)
	No	1186	27 (2.3)		1186	63 (5.3)		1186	553 (46.6)	
Chronic pulmonary disease	Yes	120	2 (1.7)	0.72 (0.17-3.07)	120	9 (7.5)	1.49 (0.72-3.09)	120	48 (40.0)	0.75 (0.51-1.10)
	No	1083	25 (2.3)		1083	56 (5.2)		1083	510 (47.1)	
Connective tissue disease	Yes	16	0 (0.0)	NA#	16	1 (6.3)	1.17 (0.15-9.00)	16	5 (31.3)	0.52 (0.18-1.51)
	No	1187	27 (2.3)		1187	64 (5.4)		1187	553 (46.6)	
Peptic ulcer/erosion	Yes	104	1 (1.0)	0.40 (0.05-2.98)	104	6 (5.8)	1.08 (0.45-2.56)	104	48 (46.2)	0.99 (0.66-1.48)
	No	1099	26 (2.4)		1099	59 (5.4)		1099	510 (46.4)	
Mild liver disease	Yes	397	4 (1.0)	0.35 (0.12-1.01)	397	16 (4.0)	0.65 (0.36-1.16)	397	200 (50.4)	1.27 (0.99-1.62)
	No	806	23 (2.9)		806	49 (6.1)		806	358 (44.4)	
Diabetes without complication	Yes	172	7 (4.1)	2.15 (0.89-5.15)	172	15 (8.7)	1.87 (1.03-3.42) *	172	91 (52.9)	1.36 (0.98-1.88)
	No	1031	20 (1.9)		1031	50 (4.9)		1031	467 (45.3)	
Hemiplegia	Yes	5	0 (0.0)	NA#	5	0 (0.0)	NA#	5	2 (40.0)	0.77 (0.13-4.62)
	No	1198	27 (2.3)		1198	65 (5.4)		1198	566 (46.4)	
Moderate or severe renal disease	Yes	38	3 (7.9)	4.08 (1.17-14.17)*	38	4 (10.5)	2.13 (0.73-6.19)	38	20 (52.6)	1.29 (0.68-2.47)
	No	1165	24 (2.1)		1165	61 (5.2)		1165	538 (46.2)	
Diabetes with complication	Yes	39	0 (0.0)	NA#	39	0 (0.0)	NA#	39	21 (53.9)	1.36 (0.72-2.58)
	No	1164	27 (2.3)		1164	65 (5.6)		1164	537 (46.1)	
Malignant tumor	Yes	76	4 (5.3)	2.67 (0.90-7.92)	76	9 (11.8)	2.57 (1.22-5.42)	76	37 (48.68)	1.10 (0.69-1.76)
	No	1127	23 (2.0)		1127	56 (5.0)		1127	521 (46.2)	
Lymphoma	Yes	1	0 (0.0)	NA#	1	0 (0.0)	NA#	1	0 (0.0)	NA#
	No	1199	27 (2.3)		1199	65 (5.4)		1199	558 (46.4)	
Leukemia	Yes	4	0 (0.0)	NA#	4	0 (0.0)	NA#	4	3 (75.0)	3.48 (0.36-33.56)
	No	1202	27 (2.3)		1202	65 (5.4)		1202	555 (46.3)	
Moderate or severe liver disease	Yes	21	3 (14.3)	8.04 (2.22-29.14)*	21	1 (4.8)	0.87 (0.12-6.61)	21	9 (42.9)	0.86 (0.36-2.07)
	No	1182	24 (2.0)		1182	64 (5.4)		1182	549 (46.5)	
Metastatic solid tumor	Yes	13	2 (15.4)	8.48 (1.78-40.23)*	13	1 (7.7)	NA#	13	4 (30.8)	0.51 (0.16-1.67)
	No	1190	25 (2.1)		1190	64 (5.4)		1190	554 (46.6)	
AIDS/HIV	Yes	0	0 (0.0)	NA	0	0 (0.0)	NA	0	0 (0.0)	NA
	No	1203	27 (2.3)		1203	65 (5.4)		1203	558 (46.4)	

Comorbidity		Local complications: Total, n	Local complications: Yes, n (%)	Local complications: OR (95% CI)	Fluid collection: Total, n	Fluid collection: Yes, n (%)	Fluid collection: OR (95% CI)	Pseudocyst: Total, n	Pseudocyst: Yes, n (%)	Pseudocyst: OR (95% CI)
Myocardial infarction	Yes	56	15 (26.8)	0.85 (0.46-1.56)	56	12 (21.4)	0.80 (0.42-1.53)	56	3 (5.4)	0.50 (0.15-1.61)
	No	1141	343 (30.1)		1141	291 (25.5)		1142	117 (10.3)	
Congestive heart failure	Yes	59	10 (17.0)	0.46 (0.23-0.93)*	59	8 (13.6)	0.45 (0.21-0.96)*	59	3 (5.1)	0.47 (0.14-1.52)
	No	1138	348 (30.6)		1138	295 (25.9)		1139	117 (10.3)	
Peripheral vascular disease	Yes	122	36 (29.5)	0.98 (0.65-1.48)	122	29 (23.8)	0.91 (0.59-1.41)	122	14 (11.5)	1.19 (0.66-2.14)
	No	1075	322 (30.0)		1075	274 (25.5)		1076	106 (9.9)	
Cerebrovascular disease	Yes	117	33 (28.2)	0.91 (0.60-1.39)	117	28 (23.9)	0.92 (0.59-1.44)	117	10 (8.6)	0.83 (0.42-1.62)
	No	1080	325 (30.1)		1080	275 (25.5)		1081	110 (10.2)	
Dementia	Yes	17	3 (17.7)	0.50 (0.14-1.74)	17	1 (5.9)	0.18 (0.02-1.38)	17	1 (5.9)	0.56 (0.07-4.24)
	No	1180	355 (30.1)		1180	302 (25.6)		1181	119 (10.1)	
Chronic pulmonary disease	Yes	120	34 (28.3)	0.92 (0.61-1.40)	120	29 (24.2)	0.93 (0.60-1.45)	120	10 (8.3)	0.80 (0.41-1.57)
	No	1077	324 (30.1)		1077	274 (25.4)		1078	110 (10.2)	
Connective tissue disease	Yes	16	4 (25.09)	0.78 (0.25-2.43)	16	3 (18.8)	0.68 (0.19-2.39)	16	3 (18.8)	2.10 (0.59-7.48)
	No	1181	354 (30.0)		1181	300 (25.4)		1182	117 (9.9)	
Peptic ulcer/erosion	Yes	104	31 (29.8)	0.99 (0.64-1.54)	104	26 (25.0)	0.98 (0.62-1.56)	104	12 (11.5)	1.19 (0.63-2.24)
	No	1093	327 (29.9)		1093	277 (25.3)		1094	108 (9.9)	
Mild liver disease	Yes	393	146 (37.2)	1.65 (1.28-2.13)*	393	121 (30.8)	1.52 (1.16-1.99)*	393	40 (10.2)	1.03 (0.69-1.53)
	No	804	212 (26.4)		804	182 (22.6)		805	80 (9.9)	
Diabetes without complication	Yes	171	56 (32.8)	1.17 (0.83-1.65)	171	54 (31.6)	1.33 (1.01-2.05)*	171	15 (8.8)	0.84 (0.48-1.49)
	No	1026	302 (29.4)		1026	249 (24.3)		1027	105 (10.2)	
Hemiplegia	Yes	5	0 (0.0)	NA	5	0 (0.0)	NA	5	0 (0.0)	NA
	No	1192	358 (30.0)		1192	303 (25.4)		1193	120 (10.1)	
Moderate or severe renal disease	Yes	38	11 (29.0)	0.95 (0.47-1.94)	38	8 (21.1)	0.78 (0.35-1.72)	38	3 (7.9)	0.76 (0.23-2.52)
	No	1159	347 (29.9)		1159	295 (25.5)		1160	117 (10.1)	
Diabetes with complication	Yes	39	8 (20.5)	0.60 (0.27-1.31)	39	6 (15.4)	0.53 (0.22-1.27)	39	5 (12.8)	1.34 (0.51-3.49)
	No	1158	350 (30.2)		1158	298 (25.7)		1159	115 (9.9)	
Malignant tumor	Yes	76	24 (31.6)	1.09 (0.66-1.79)	76	21 (27.6)	1.14 (0.68-1.91)	76	6 (7.9)	0.76 (0.32-1.78)
	No	1121	334 (29.8)		1121	282 (25.2)		1122	114 (10.2)	
Lymphoma	Yes	1	0 (0.0)	NA	1	0 (0.0)	NA	1	0 (0.0)	NA
	No	1196	358 (29.9)		1196	303 (25.3)		1197	120 (10.0)	
Leukemia	Yes	4	1 (25.0)	0.78 (0.08-7.53)	4	1 (25.0)	0.98 (0.10-9.49)	4	1 (25.0)	3.01 (0.31-29.18)
	No	1193	357 (29.9)		1193	302 (25.3)		1194	119 (10.0)	
Moderate or severe liver disease	Yes	21	5 (23.8)	0.73 (0.26-2.00)	21	3 (14.3)	0.49 (0.14-1.66)	21	0 (0.0)	NA
	No	1176	353 (30.0)		1176	300 (25.5)		1177	120 (10.2)	
Metastatic solid tumor	Yes	13	4 (30.8)	1.04 (0.32-3.41)	13	4 (30.8)	1.32 (0.40-4.30)	13	1 (7.7)	0.75 (0.10-5.79)
	No	1184	354 (29.9)		1184	299 (25.3)		1185	119 (10.0)	
AIDS/HIV	Yes	0	0 (0.0)	NA	0	0 (0.0)	NA	0	0 (0.0)	NA
	No	1203	358 (29.9)		1197	303 (25.3)		1198	120 (10.2)	

Comorbidity		Necrosis: Total, n	Necrosis: Yes, n (%)	Necrosis: OR (95% CI)	Systemic complications: Total, n	Systemic complications: Yes, n (%)	Systemic complications: OR (95% CI)	Respiratory failure: Total, n	Respiratory failure: Yes, n (%)	Respiratory failure: OR (95% CI)
Myocardial infarction	Yes	56	4 (7.1)	0.74 (0.26-2.10)	55	7 (12.7)	1.81 (0.79-4.12)	55	7 (12.7)	3.31 (1.43-7.71)*
	No	1142	107 (9.4)		1140	85 (7.5)		1139	48 (4.2)	
Congestive heart failure	Yes	59	1 (1.7)	0.16 (0.02-1.18)	57	9 (15.8)	2.38 (1.13-5.03)*	57	7 (12.3)	3.18 (1.37-7.37)*
	No	1139	110 (9.7)		1138	83 (7.3)		1137	48 (4.2)	
Peripheral vascular disease	Yes	122	11 (9.0)	0.97 (0.50-1.86)	120	17 (14.2)	2.20 (1.25-3.87)*	120	11 (9.2)	2.36 (1.19-4.71)*
	No	1076	100 (9.3)		1075	75 (7.0)		1074	44 (4.1)	
Cerebrovascular disease	Yes	117	9 (7.7)	0.80 (0.39-1.63)	116	20 (17.2)	2.91 (1.7-4.99)*	116	13 (11.2)	3.11 (1.62-5.99)*
	No	1081	102 (9.4)		1079	72 (6.7)		1078	42 (3.9)	
Dementia	Yes	17	2 (11.8)	1.31 (0.30-5.81)	17	2 (11.8)	1.61 (0.36-7.16)	17	2 (11.8)	2.83 (0.63-12.68)
	No	1181	109 (9.2)		1178	90 (7.6)		1177	53 (4.5)	
Chronic pulmonary disease	Yes	120	9 (7.5)	0.78 (0.38-1.58)	119	15 (12.6)	1.87 (1.04-3.37)*	119	13 (10.9)	3.02 (1.57-5.80)*
	No	1078	102 (9.5)		1076	77 (7.2)		1075	42 (3.9)	
Connective tissue disease	Yes	16	2 (12.5)	1.41 (0.32-6.27)	16	2 (12.5)	1.73 (0.38-7.72)	16	2 (12.5)	3.03 (0.67-13.69)
	No	1182	109 (9.2)		1179	90 (7.6)		1178	53 (4.5)	
Peptic ulcer/erosion	Yes	104	8 (7.7)	0.80 (0.38-1.70)	103	8 (7.8)	1.01 (0.47-2.15)	102	5 (4.9)	1.07 (0.42-2.76)
	No	1094	103 (9.4)		1092	84 (7.7)		1092	50 (4.6)	
Mild liver disease	Yes	393	51 (13.0)	1.86 (1.25-2.75)*	392	24 (6.12)	0.70 (0.44-1.14)	392	12 (3.1)	0.56 (0.29-1.07)
	No	805	60 (7.5)		803	68 (8.47)		802	43 (5.4)	
Diabetes without complication	Yes	171	16 (9.4)	1.01 (0.58-1.77)	172	21 (12.2)	1.87 (1.11-3.12)*	172	14 (8.1)	2.12 (1.13-3.98)*
	No	1027	95 (9.3)		1023	71 (6.9)		1022	41 (4.0)	
Hemiplegia	Yes	5	0 (0.0)	NA	5	1 (20.0)	3.02 (0.33-27.30)	5	0 (0.0)	NA
	No	1193	111 (9.3)		1190	91 (7.7)		1194	55 (4.6)	
Moderate or severe renal disease	Yes	38	3 (7.9)	0.83 (0.26-2.76)	38	6 (15.8)	2.34 (0.95-5.74)	38	2 (5.3)	1.16 (0.27-4.93)
	No	1160	108 (9.3)		1157	86 (7.4)		1156	53 (4.6)	
Diabetes with complication	Yes	39	0 (0.0)	NA (p=0.0436)	39	1 (2.6)	0.31 (0.04-2.27)	39	1 (3.6)	0.54 (0.07-3.98)
	No	1159	111 (9.6)		1156	91 (7.9)		1155	54 (4.7)	
Malignant tumor	Yes	76	5 (6.6)	0.68 (0.27-1.71)	76	9 (11.8)	1.68 (0.81-3.48)	76	7 (9.2)	2.26 (0.99-5.18)
	No	1122	106 (9.5)		1119	83 (7.4)		1118	48 (4.3)	
Lymphoma	Yes	1	0 (0.0)	NA	1	0 (0.0)	NA	1	0 (0.0)	NA
	No	1197	111 (9.3)		1194	92 (7.7)		1193	55 (4.6)	
Leukemia	Yes	4	0 (0.0)	NA	4	1 (25.0)	4.03 (0.41-39.13)	4	1 (25.0)	7.01 (0.72-68.53)
	No	1194	111 (9.3)		1191	91 (7.6)		1190	54 (4.5)	
Moderate or severe liver disease	Yes	21	0 (0.0)	NA	21	4 (19.1)	2.90 (0.96-8.82)	21	2 (9.5)	2.22 (0.50-9.80)
	No	1177	111 (9.4)		1174	88 (7.5)		1173	53 (4.5)	
Metastatic solid tumor	Yes	13	0 (0.0)	NA	13	2 (15.4)	2.21 (0.48-10.11)	13	1 (7.7)	1.74 (0.22-13.62)
	No	1185	111 (9.4)		1182	90 (7.6)		1181	54 (4.6)	
AIDS/HIV	Yes	0	0 (0.0)	NA	0	0 (0.0)	NA	0	0 (0.0)	NA
	No	1198	111 (9.4)		1195	92 (7.7)		1194	(4.6)	

Comorbidity		Heart failure: Total, n	Heart failure: Yes, n (%)	Heart failure: OR (95% CI)	Renal failure: Total, n	Renal failure: Yes, n (%)	Renal failure: OR (95% CI)
Myocardial infarction	Yes	55	1 (1.8)	1.15 (0.15-8.81)	55	1 (1.8)	0.64 (0.09-4.78)
	No	1140	18 (1.6)		1140	32 (2.8)	
Congestive heart failure	Yes	57	4 (7.0)	5.65 (1.81-17.6)*	57	3 (5.3)	2.05 (0.61-6.94)
	No	1138	15 (1.3)		1138	30 (2.6)	
Peripheral vascular disease	Yes	120	7 (5.8)	5.49 (2.1-14.2)*	120	6 (5.0)	2.04 (0.83-5.05)
	No	1075	12 (1.1)		1075	27 (2.5)	
Cerebrovascular disease	Yes	116	3 (2.6)	1.76 (0.51-6.15)	116	7 (6.0)	2.60 (1.10-6.13)*
	No	1079	16 (1.5)		1079	26 (2.4)	
Dementia	Yes	17	0 (0.0)	NA	17	0 (0.0)	NA
	No	1178	19 (1.6)		1178	33 (2.8)	
Chronic pulmonary disease	Yes	119	3 (2.5)	1.71 (0.49-5.97)	119	2 (1.7)	0.58 (0.14-2.44)
	No	1076	16 (1.5)		1076	31 (2.9)	
Connective tissue disease	Yes	16	0 (0.0)	NA	16	0 (0.0)	NA
	No	1179	19 (1.6)		1179	33 (2.8)	
Peptic ulcer/erosion	Yes	103	2 (1.9)	1.25 (0.29-5.50)	103	2 (1.9)	0.68 (0.16-2.87)
	No	1092	17 (1.6)		1092	31 (2.8)	
Mild liver disease	Yes	392	3 (0.8)	0.38 (0.11-1.31)	392	9 (2.3)	0.76 (0.35-1.66)
	No	803	16 (2.0)		803	24 (3.0)	
Diabetes without complication	Yes	172	4 (2.3)	1.60 (0.52-4.88)	172	8 (4.7)	1.95 (0.86-4.39)
	No	1023	15 (1.5)		1023	25 (2.4)	
Hemiplegia	Yes	5	0 (0.0)	NA	5	1 (20.0)	9.05 (0.98-83.24)
	No	1190	19 (1.6)		1190	32 (2.7)	
Moderate or severe renal disease	Yes	38	1 (2.6)	1.71 (0.22-13.15)	38	5 (13.2)	6.11 (2.2-16.82)*
	No	1157	18 (1.6)		1157	28 (2.4)	
Diabetes with complication	Yes	39	0 (0.0)	NA	39	0 (0.0)	NA
	No	1156	19 (1.6)		1156	33 (2.9)	
Malignant tumor	Yes	76	4 (5.3)	4.09 (1.32-12.64)*	76	5 (6.6)	2.74 (1.03-7.32)
	No	1119	15 (1.3)		1119	28 (2.5)	
Lymphoma	Yes	1	0 (0.0)	NA	1	0 (0.0)	NA
	No	1194	19 (1.6)		1194	33 (2.8)	
Leukemia	Yes	4	0 (0.0)	NA	4	0 (0.0)	NA
	No	1191	19 (1.6)		1191	33 (2.8)	
Moderate or severe liver disease	Yes	21	2 (9.5)	7.16 (1.55-33.21)*	21	2 (9.5)	3.88 (0.87-17.40)
	No	1174	17 (1.5)		1174	31 (2.6)	
Metastatic solid tumor	Yes	13	1 (7.7)	5.39 (0.66-43.68)	13	1 (7.7)	2.99 (0.38-23.73)
	No	1182	18 (1.5)		1182	32 (2.7)	
AIDS/HIV	Yes	0	0 (0.0)	NA	0	0 (0.0)	NA
	No	1195	19 (1.6)		1195	33 (2.7)	
