# Supplementary material for: Aging and Comorbidities in Acute Pancreatitis II.: A Cohort-Analysis of 1203 Prospectively Collected Cases
Source: Front Physiol. 2019 Apr 2;9:1776. doi: 10.3389/fphys.2018.01776 (PMC6454835; doi:10.3389/fphys.2018.01776)
Supplement: APPENDIX 10 — Results of multivariate analysis on the effects of individual comorbidities on the outcomes of acute pancreatitis. [file Data_Sheet_10.PDF]

Supplementary Appendix 10. Results of multivariate analysis on the effects of indivdiual comorbidities on the outcomes of acute pancreatitis

	Local complications			Fluid collection			Pseudocyst			Necrosis		
	β	OR (95% CI)	p	β	OR (95% CI)	p	β	OR (95% CI)	p	β	OR (95% CI)	p
Age categories												
18-34 y (young adults)	0	1 (ref)		0	1 (ref)		0	1 (ref)		0	1 (ref)	
35-64 y (middle-aged adults)	0.74	2.11 (1.29-3.45)*	0.003*	0.58	1.79 (1.07-2.98)*	0.026*	1.21	3.34 (1.31-8.53)*	0.012*	1.14	3.14 (1.23-8.01)*	0.017*
> 65 y (old adults)	0.40	1.49 (0.88-2.54)	0.136	0.31	1.36 (0.79-2.35)	0.266	0.91	2.48 (0.93-6.59)	0.069#	0.60	1.83 (0.67-4.98)	0.237
Comorbidity categories												
Myocardial infarction	0.02	1.02 (0.53-1.96)	0.944	-0.04	0.97 (0.48-1.94)	0.921	-0.63	0.54 (0.16-1.82)	0.314	0.09	1.09 (0.37-3.24)	0.879
Congestive heart failure	-0.68	0.51 (0.24-1.06)	0.071#	-0.70	0.50 (0.22-1.11)	0.086#	-0.62	0.53 (0.16-1.82)	0.319	-1.70	0.18 (0.02-1.39)	0.100
Peripheral vascular disease	0.14	1.15 (0.74-1.80)	0.528	0.04	1.04 (0.68-1.78)	0.884	0.38	1.46 (0.77-2.76)	0.239	0.31	1.37 (0.68-2.75)	0.383
Cerebrovascular disease	0.06	1.06 (0.67-1.68)	0.808	0.10	1.10 (0.68-1.78)	0.695	-0.08	0.92 (0.45-1.88)	0.816	-0.06	0.95 (0.44-2.03)	0.885
Dementia	-0.50	0.61 (0.17-2.20)	0.446	-1.63	0.20 (0.03-1.53)	0.200	-0.40	0.67 (0.08-5.32)	0.704	0.75	2.11 (0.44-10.19)	0.351
Chronic pulmonary disease	-0.10	0.90 (0.58-1.40)	0.648	-0.06	0.94 (0.59-1.49)	0.795	-0.22	0.80 (0.40-1.61)	0.536	-0.20	0.82 (0.39-1.72)	0.604
Connective tissue disease	-0.35	0.71 (0.22-2.28)	0.561	-0.44	0.64 (0.18-2.33)	0.501	0.82	2.28 (0.62-8.38)	0.215	0.45	1.57 (0.34-7.28)	0.567
Peptic ulcer/erosion	-0.05	0.96 (0.61-1.50)	0.840	-0.05	0.95 (0.59-1.53)	0.827	0.14	1.15 (0.60-2.19)	0.674	-0.26	0.77 (0.36-1.65)	0.502
Mild liver disease	0.39	1.47 (1.12-1.93)*	0.005*	0.28	1.32 (0.99-1.76)	0.053		.			-	
Diabetes without complication	0.03	1.03 (0.72-1.48)	0.869	0.30	1.34 (0.93-1.94)	0.116		-			-	
Hemiplegia	-13.39	NA	0.982	-13.23	NA	0.984		-			-	
Moderate or severe renal disease	0.21	1.23 (0.58-2.63)	0.591	-0.02	0.98 (0.43-2.50)	0.958	-0.14	0.87 (0.26-2.95)	0.820	0.12	1.12 (0.33-3.85)	0.852
Diabetes with complication	-0.55	0.58 (0.25-1.33)	0.197	-0.58	0.56 (0.22-1.40)	0.214		-			-	
Malignant tumor	0.11	1.12 (0.67-1.88)	0.672	0.19	1.21 (0.71-2.08)	0.480		-			-	
Lymphoma	-13.00	NA	0.992	-12.84	NA	0.991		-			-	
Leukemia	-0.21	0.81 (0.08-8.14)	0.861	-0.01	0.99 (0.10-9.82)	0.990		-			-	
Moderate or severe liver disease	-0.28	0.75 (0.27-2.11)	0.589	-0.74	0.48 (0.14-1.66)	0.245		-			-	
Metastatic solid tumor	0.07	1.07 (0.32-3.58)	0.914	0.37	1.45 (0.43-4.88)	0.544		-			-	
Any tumors		-			-		-0.26	0.77 (0.36-1.66)	0.503	-0.67	0.51 (0.201-1.32)	0.165
Liver disease at any stage		-			-		-0.18	0.84 (0.55-1.27)	0.400	0.44	1.56 (1.03-2.35)*	0.035*
Diabetes with or without complications		-			-		-0.03	0.97 (0.57-1.64)	0.906	-0.35	0.70 (0.40-1.25)	0.230

	Systemic complications			LOH		
	β	OR (95% CI)	p	β	OR (95% CI)	p
Age categories						
18-34 y (young adults)	0	1 (ref)		0	1 (ref)	
35-64 y (middle-aged adults)	2.15	8.55 (1.16-63.03)	0.035*	0.62	1.85 (1.22-2.81)	0.004*
> 65 y (old adults)	2.03	7.60 (1.02-56.93)	0.048*	0.43	1.53 (0.98-2.39)	0.061
Comorbidity categories						
Myocardial infarction	0.00	1.00 (0.40-2.51)	0.998	0.80	2.24 (1.22-4.10)	0.009*
Congestive heart failure	0.34	1.40 (0.61-3.21)	0.422	0.21	1.28 (0.69-2.21)	0.472
Peripheral vascular disease	0.39	1.48 (0.80-2.72)	0.214	0.02	1.02 (0.68-1.54)	0.926
Cerebrovascular disease	0.83	2.29 (1.29-4.07)	0.005*	0.09	1.10 (0.73-1.66)	0.662
Dementia		-		-0.78	0.46 (0.15-1.37)	0.162
Chronic pulmonary disease	0.35	1.42 (0.77-2.63)	0.265	-0.39	0.68 (0.45-1.01)	0.057#
Connective tissue disease		-		-0.77	0.47 (0.16-1.38)	0.168
Peptic ulcer/erosion		-		0.0	1.00 (0.66-1.51)	0.984
Mild liver disease		-		0.17	1.18 (0.92-1.53)	0.198
Diabetes without complication		-		0.18	1.20 (0.86-1.69)	0.287
Hemiplegia		-		-0.19	0.83 (0.13-5.13)	0.839
Moderate or severe renal disease	0.51	1.66 (0.65-4.27)	0.292	0.12	1.13 (0.57-2.26)	0.728
Diabetes with complication		-		0.15	1.16 (0.59-2.30)	0.666
Malignant tumor		-		0.09	1.09 (0.68-1.77)	0.714
Lymphoma		-		-12.32	NA	0.978
Leukemia		-		1.19	3.06 (0.31-30.04)	0.337
Moderate or severe liver disease		-		-0.12	0.89 (0.36-2.17)	0.795
Metastatic solid tumor		-		-0.62	0.54 (0.16-1.79)	0.312
Any tumors	0.40	1.49 (0.77-2.89)	0.240		-	
Liver disease at any stage	-0.33	0.72 (0.44-1.17)	0.180		-	
Diabetes with or without complications	0.20	1.22 (0.72-2.09)	0.470		-	

	Mortality		
	β	OR (95% CI)	p
Age categories			
18-34 y (young adults)	-12.51	NA	0.966
35-64 y (middle-aged adults)	-0.42	0.66 (0.30-1.46)	0.303
> 65 y (old adults)	0	1 (ref)	
Comorbidity categories			
Moderate or severe renal disease	1.35	3.86 (1.07-13.88)	0.004*
Moderate or severe liver disease	2.09	8.12 (2.13-20.96)	0.002*
Metastatic tumors	1.93	6.90 (1.36-34.88)	0.020*

	Severity		
	β	OR (95% CI)	p
Age categories			
18-34 y (young adults)	0	1 (ref)	
35-64 y (middle-aged adults)	-0.09	0.92 (0.53-1.59)	0.755
> 65 y (old adults)	1.82	6.20 (0.81-47.18)	0.078
Comorbidity categories			
Malignant tumors	-0.68	0.51 (0.25-1.04)	0.065#
Liver diseases	0.55	1.73 (0.96-3.12)	0.066#
Diabetes	-0.30	0.74 (0.42-1.39)	0.335
Cardiovascular diseases	-0.27	0.77 (0.42-1.39)	0.383
Pulmonary diseases	-0.20	0.82 (0.39-1.74)	0.602
